# Theory of cell fate

**DOI:** 10.1002/wsbm.1471

**Published:** 2019-12-12

**Authors:** Michael J. Casey, Patrick S. Stumpf, Ben D. MacArthur

**Affiliations:** ^1^ Mathematical Sciences University of Southampton Southampton UK; ^2^ Institute for Life Sciences University of Southampton Southampton UK; ^3^ Centre for Human Development, Stem Cells and Regeneration, Faculty of Medicine University of Southampton Southampton UK

**Keywords:** cell fate, mathematical model, systems biology

## Abstract

Cell fate decisions are controlled by complex intracellular molecular regulatory networks. Studies increasingly reveal the scale of this complexity: not only do cell fate regulatory networks contain numerous positive and negative feedback loops, they also involve a range of different kinds of nonlinear protein–protein and protein–DNA interactions. This inherent complexity and nonlinearity makes cell fate decisions hard to understand using experiment and intuition alone. In this primer, we will outline how tools from mathematics can be used to understand cell fate dynamics. We will briefly introduce some notions from dynamical systems theory, and discuss how they offer a framework within which to build a rigorous understanding of what we mean by a cell “fate”, and how cells change fate. We will also outline how modern experiments, particularly high‐throughput single‐cell experiments, are enabling us to test and explore the limits of these ideas, and build a better understanding of cellular identities.

This article is categorized under:

Models of Systems Properties and Processes > Mechanistic Models
Biological Mechanisms > Cell Fates
Models of Systems Properties and Processes > Cellular Models

## Graphical abstract

The cell as a dynamical system: combining theory and experiment to understand cell fates.

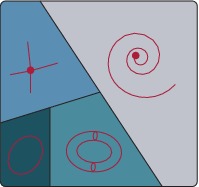

## 1 INTRODUCTION

Cells in multicellular organisms typically specialize into distinct “types”, which perform specific functions within the context of the tissue, and organism, within which they reside. The concept of cell “type” has many definitions, reflecting the long history of cell biology and accumulation of experimental methods (Clevers et al., 2017). Cells were first discovered with the advent of microscopy (Hooke, 2003), and following the naturalist tradition of the time, were organized into distinct classes by morphology and function. How these classes arise has been a longstanding question.

An early idea was that each distinct cell “type” carries only part of the genome and therefore has access to a different set of “instructions” of how to behave. This idea, although appealing, was proven false by pioneering experiments of Gurdon in the 1970s who showed, via serial transplantation of the nuclear content of adult cells into enucleated eggs and monitoring their development, that individual adults cells possess the organism's entire genome (Laskey & Gurdon, 1970). We now know that essentially all adult cells within an organism possess the same genome (aside from certain lymphocytes, some neurons and anuclear cells, such as mature red blood cells and platelets), yet different cells express this genome in different ways.

From the purely biological point‐of‐view, this is the de facto working definition of a cell type: different cell types are defined by the different genes and proteins that they express, and the different functions they accordingly perform. This working definition has motivated much experimental work because it provides a convenient way to associate cellular functions with particular patterns of molecular expression. For example, different types of stem and progenitor cells are routinely characterized by the different combinations of cell surface markers that they express (Akashi, Traver, Miyamoto, & Weissman, 2000; Lv, Tuan, Cheung, & Leung, 2014; Thomson et al., 1998).

This simple, flexible definition of cell type has been disrupted with the advent of high‐throughput, single‐cell methods, primarily single‐cell RNA‐sequencing (scRNA‐seq) which have revealed substantial “heterogeneity” within previously established cell types (Björklund et al., 2016; Buettner et al., 2015; Grün et al., 2015; Zeisel et al., 2015). In this context, heterogeneity is typically taken to mean that cells of the same type can have substantially different levels of gene expression, including in genes that may have been used to define that cell type. Increasingly it is becoming clear that traditional ways of identifying and categorizing cell types are unable to capture this complexity, leading to ambiguity as to what is really meant by a cell type. A rigorous, consistent definition of cell type, is therefore required to make use of this new wave of gene expression data. By developing such a theory, we can begin to address the question of *why* patterns of expression, and not some others, occur; or why particular patterns of expression should confer particular functions to the cell.

These “why” questions hint that there is a deeper truth to be found concerning the rules that govern how the genome is outworked in a cell, beyond simple cataloguing of which cells express which factors. This is a complex question that is difficult to address in a purely experimental way. However, mathematical models provide a logical framework within which to explore complex issues like this, and there has accordingly been a long and fruitful history of biologists and theoreticians working together to explore this issue theoretically, and wrestling with how mathematical notions relate to the complexity observed in experiment.

Here, we will outline some of the main lines of thought that have emerged from this discussion. In the first part of this primer, we will discuss some theoretical notions before exploring how these notions relate to experiment.

## 2 CELL FATE: IN THEORY

To begin our discussion, it is useful to distinguish between the *state* of a cell and its *fate*. Before we start it is worth noting that the terms cell “type” and cell “fate” are often used interchangeably. As a technicality, however, fate refers to the future of the cell—the “type” toward which it is progressing. For reasons that will become apparent, we will focus on cell fates specifically.

For the purposes of our discussion we will assume that there are a set of molecular identifiers (or species) *X*_*i*_ that and individual cell may (or may not) express. For example, *X*_*i*_ may denote the abundance of the mRNA product of the *i*th gene in the genome, or the abundance of the *i*th protein in the proteome. The complete molecular state of a cell can then be encoded in the vector ***x***(*t*) = *x*_*i*_(*t*) ∈ *X*, where *x*_*i*_(*t*) is the abundance of the *i*th molecular species at time *t* and *X* denotes the space of all possible expression patterns. It is important to note that the state of the cell may (in fact, certainly will) change over time, and so we make this explicit in our formalism from the start. It is also notable that a given experimental method will inevitably only measure a subset of the molecular identifiers that specify a cell, so the definition of a cell state is contextual and will depend on the experimental method used.

**Figure 1:**
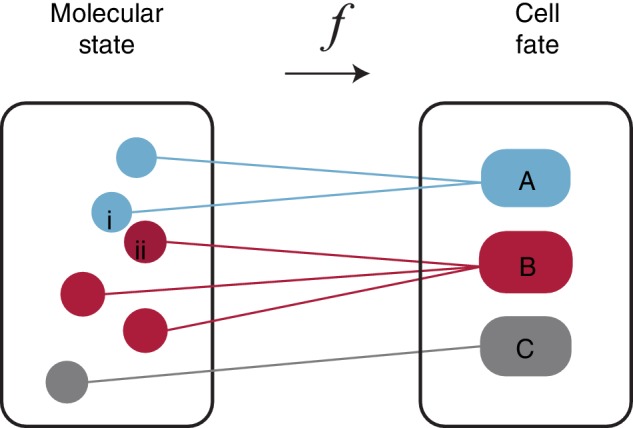
Mapping molecular states to cell fates. An individual cell can be described both in terms of its molecular state, and its fate. Each point in the molecular state box is a complete descriptor of the molecular constitution of a cell. The mapping between states and fates is many‐to‐one: different subsets of expression state space may map to the same fates. Here, three different fates A, B, and C, are illustrated, colored blue, red, and gray. Furthermore, similar molecular expression states may map to different fates. Two such states, marked *i* and *ii* are illustrated

We now wish to establish how these cell states relate to distinct cellular functions or fates (also known as types—henceforth we will use the term cell fate, for clarity of nomenclature) in the adult or developing organism. To do so, we will assume that there is a mapping from cell states to cell fates (Figure 1). To construct this map, let *y*_*i*_ ∈ *Y* denote the *i*th cell fate and *Y* denote the set of all possible fates. Traditionally, there were thought to be 200–300 distinct cell fates in the adult human (Junqueira, Junqueira, Carneiro, & Kelley, 1992) (although by uncovering hitherto unappreciated heterogeneity within established cell types recent efforts, such as the Human Cell Atlas, are revising this estimate (Lukowski et al., 2019; Regev et al. (2017)). By contrast, the human genome contains approximately 20,000 genes (Church et al., 2009), each of which may be expressed at varying levels. Even if each gene can only be expressed at two levels (i.e., “on” or “off”) this gives ∼2^20,000^ distinct cell states. Thus, from purely mathematical reasoning the number of possible cell states vastly outnumbers the number of distinct cellular functions. It is clear, therefore, that not all molecular states can map to distinct cell fates. From a mathematical perspective this means that our mapping from cell states to cell fates is many‐to‐one (Figure 1).

This simple observation has a couple of important consequences. First, it means that many different cell states may map to the same fate. Mathematicians would say that the mapping is surjective (assuming the biologically reasonable technicality that every state maps to *some* function). Biologically this means that slight variations the molecular expression patterns will not typically affect cell function. For example, an extra copy of a housekeeping protein is unlikely to significantly alter the behavior of a cell; neither is the loss of a single copy of an abundantly expressed mRNA. The mathematical statement of surjectivity therefore relates to the biological fact that molecular expression patterns are likely to fluctuate in the cell, while the identity or function of the cell will typically be robust to these fluctuations. However, some fluctuations must result in a change in identity, or there would only be one possible cell fate. Thus, there must be some cell states that are “close” to each other, yet nevertheless map to different fates (how we chose to define “close” is important here, and an issue that we will discuss soon). This means that the mapping must have some structure and, in particular, there must be “fault lines” that separate *X* into discrete pieces $X_{i}$, each of which maps to a different fate *y*_*i*_ (as a mathematical technicality, each $X_{i}$ need not be connected). To understand this structure that emerges in the mapping between genotype and phenotype, it is helpful to take a short detour into some dynamical systems theory.

In its most general form, a continuous dynamical system is a set of coupled ordinary differential equations that describe how a set of time‐dependent variables *x*_*i*_(*t*), for *i* = 1, 2, … *n*, evolve over time:

(1) $$\frac{dx}{\mathrm{dt}}=F\left(x\right),$$

where ***F***(***x***) is a set of coupling functions that encode the way in which the rates of change of the *x*_*i*_s depend on each other (without loss of generality we may assume that these coupling functions do not depend explicitly on time since we can always set *x*_*n* + 1_(*t*) = *t* to put the equations in the form above). If *x*_*i*_(*t*) is associated with the expression of the *i*th molecular species in the cell, then this dynamical system describes how molecular expression patterns evolve over time. If Equation (1) describes a system that is out of thermodynamic equilibrium (i.e., is exchanging matter and/or energy with its environment), which is the case for the cell, then the dynamical system is said to be dissipative.

**Figure 2:**
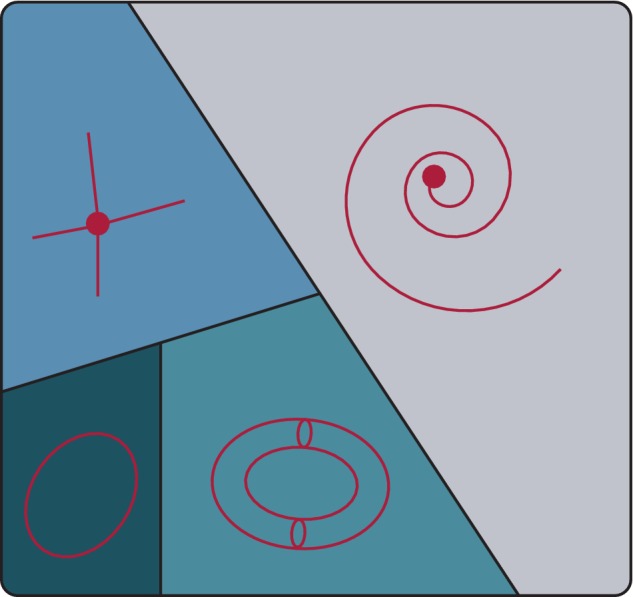
The cell as a dynamical system. Functional associations between molecular components in the cell give rise to a complex intracellular regulatory network. This network encodes the architecture of a complex dynamical system that may admit numerous attractors, each of which may be identified with a distinct cell “fate”. The basins of attraction of the attractors partition the state space into discrete pieces. The cell's intracelluar molecular dynamics may admit many different kinds of attractor including various different kinds of fixed‐point such as (Upper‐Left) stable nodes and (Right) stable spirals, as well as (Lower‐Left) limit cycles, and (Lower‐Centre) more exotic structures such as limit tori (shown) or even strange attractors

This view of the cell as a dissipative dynamical system is useful for understanding the structure of the mapping from genotype to phenotpye. One particular notion—that of the attractor—is particularly important. Although the formal definition of an attractor is subtle (Strogatz, 2018), informally an attractor of a dissipative dynamical system is an isolated subset of the state space *A* ∈ *X* toward which the system will evolve for a subset of initial conditions *X*_*A*_ ∈ *X*. The subset of initial conditions that converge toward *A* is known as the basin of attraction of *A*. Importantly, a complex dynamical system may exhibit numerous coexisting attractor states. In this case, the basins of attraction partition the state space into discrete pieces. This notion is illustrated schematically in Figure 2.

This piece of dynamical systems theory offers us a simple way to interpret the structure of the mapping from genotype to phenotype in a general way, as follows:

The intracellular regulatory networks that control cell fates encode a complex dynamical system that admits numerous coexisting attractors. Each of these attractors constitutes a distinct cell fate.

We note briefly that formally attractors are never actually attained (for any initial conditions off the attractor); they are limiting sets toward which trajectories in the dynamical system are drawn as *t* → ∞, that is, they are the ultimate “fate” of a trajectory. For this reason, attractors are better associated with cell fates rather than cell types.

This idea was originally proposed in embryonic form by Conrad Waddington in the 1930s, and was developed by Max Delbrück in the 1940s and Stuart Kauffman from the 1960s onwards (Delbrcük, 1949; Kauffman, 1969; Waddington, 1939). Again this is a simple statement that has significant consequences. First, there are many different types of attractor that can occur, including: stable stationary states (isolated points in state space *X* which represent fixed patterns of gene expression, which can be categorized into subcategories such as stable nodes, stable spirals, etc.); stable limit cycles (closed trajectories in state space, which represent self‐sustaining patterns of oscillation); as well as more exotic structures such as limit tori and strange attractors (which represent more complex rhythmic dynamics). Thus, cell fates may be associated with stable unchanging patterns of gene expression or may be characterized by stable recurrent dynamics of various different kinds.

Second, not all molecular configurations are the same: any molecular configuration that is not part of an attractor will be an unstable transient that the cell will only adopt briefly without revisit; conversely molecular configurations that are part of an attractor are “robust” in the sense that all initial condition within the basin of attraction will converge toward them over time.

Before discussing the experimental implications of this dynamical systems view, it is worth noting that this perspective is a generalization of a view that is well known to biologists: Waddington's epigenetic landscape (Waddington, 2014).

Waddington conceived a view of the dynamics of development as analogous to a ball rolling under the influence of gravity down a complex landscape, with numerous hills and valleys. As development occurs the ball, which represents the cell, takes an increasingly constrained trajectory as it is guided along valley floors that may successively divide until it ultimately comes to rest at a local minimum of the landscape.

Waddington summarized his view as follows (Waddington, 2014):

This ‘landscape’ presents, in the form of a visual model, a description of the general properties of a complicated developing system in which the course of events is controlled by many different processes that interact in such a way that they tend to balance each other …

and elsewhere (Waddington, 1939)

The line followed by the process [development] is the bottom of a valley… One might roughly say that all these genes correspond to the geological structure which moulds the form of the valley.

It is clear from these quotes that Waddington had in mind that each cell fate is what we would now call a fixed‐point attractor, and that the dynamics are governed by a process of steepest descent on a landscape, the structure of which is determined by interactions between the genes.

**Figure 3:**
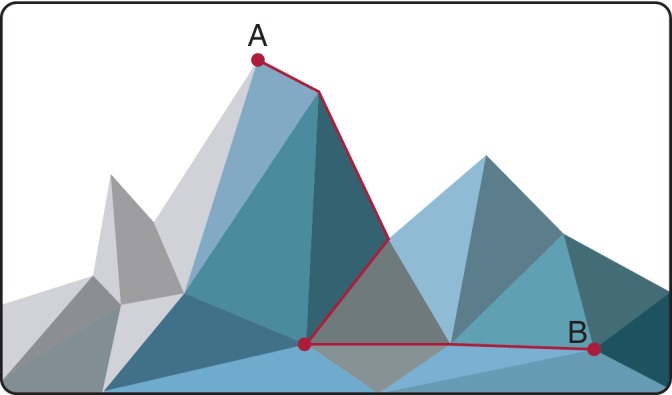
Cell fate trajectories envisaged as a path of steepest descent over a Waddington‐like landscape. The distance from molecular state A to point molecular state B over the landscape is not the same as the Euclidean distance between them in the expression space

This intuition can be encoded mathematically. To do so we first introduce a scalar potential *V*(***x***), which represents the “height” of the landscape at point ***x***. Mathematically, this potential defines the structure of an *n*‐dimensional manifold (essentially, a smooth structure that is locally equivalent to *n*‐dimensional Euclidean space; Figure 3). To account for the downhill flow, we assume that the vector field *F*(***x***) on the right‐hand side of Equation (1) can be written in terms of the gradient of this potential, and that inertia is negligible. In this case, the dynamics may be written as

(2) $$\frac{dx}{\mathrm{dt}}=-\nabla V\left(x\right),$$

where ∇ is the gradient operator.

Equation (2) is the mathematical statement of Waddington's intuition (and incidentally the equation of motion for a charged particle within an electrostatic potential *V*(***x***) neglecting inertia). It is remarkable that at the time that Waddington first made this speculation (the late 1930s) very little was known about the genes, and the theoretical foundations of dynamical systems theory had not yet been laid, yet his vision for cell fate dynamics is notably close to our current understanding.

Nevertheless, although prescient, we now know that Waddington's vision was not complete. In particular, most dynamical systems cannot be written in the form given in Equation (2) because most vector fields cannot be written as the gradient of a scalar potential: indeed this is a very strong statement, which ensures that the dynamics only admit fixed point attractors and is not generally satisfied by cell fate regulatory dynamics. For example, limit cycles corresponding to stable self‐sustaining oscillations are ubiquitous in cell biology (Abranches et al., 2014; Dunlap, 1999; Imayoshi et al., 2013; Kruse & Jülicher, 2005; Manning et al., 2019; Tyson, Chen, & Novak, 2001), yet they are not admitted by the mathematical formalism of Waddington's landscape, as given by Equation (2). In fact, Equation (2) implies the biologically unfeasible constraint that the intracellular regulatory network that defines the landscape is symmetric—that is, each regulatory edge in the network is bidirectional (Weinreb, Wolock, Tusi, Socolovsky, & Klein, 2018)—which is certainly not true.

Despite this limitation, Waddington's notion that dynamics are guided by a “landscape” that is shaped by regulatory interactions between the genes continues to be the most widely adopted understanding of cell fate dynamics. Importantly, these ideas are not just of theoretical interest. Increasingly, they are being used to guide the design and interpretation of experimental studies. In the next section, we will therefore explore how the theoretical ideas discussed in this section are used practically to decipher experimental data.

## 3 CELL FATE: IN PRACTICE

Modern experimental methods are able to profile the expression of thousands of molecular markers in thousands of individual cells in a single experiment (Cheung and Utz (2011); Macosko et al., 2015; Picelli et al., 2014). The datasets that these high‐throughput single‐cell experiments produce are allowing us to explore our understanding of the relationship between molecular expression patterns and cell fates in ever‐increasing detail. However, they can be highly complex, and require specialized computational tools to make sense of them. There is, therefore, now a burgeoning field of single‐cell analytics that aims to develop mathematical and computational methods specifically designed to dissect the structure of single‐cell data, and relate observed structures to theoretical notions of cell fate (Cao et al., 2019; Moffitt et al., 2018; Rodriques et al., 2019).

## 4 CLUSTERING AND CELL FATES

In a statistical sense, a single‐cell profiling experiment corresponds to a sampling of the space of all possible expression patterns, *X*. Because interactions between genes and proteins result in high‐dimensional associations between gene expression patterns, these samples are not uniform in *X*, but rather typically exhibit a complex clustering structure that indirectly reveals the nature of the underlying regulatory principles.

**Figure 4:**
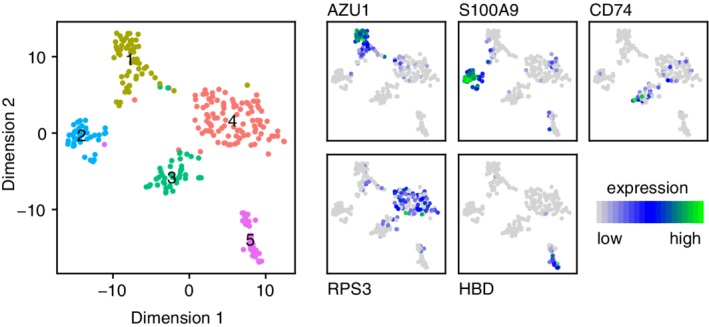
Analysis of single‐cell RNA‐Seq profiling of a human bone marrow sample. Each point is an individual cell. (Left) Data are projected into two dimensions using t‐distributed stochastic neighbor embedding (t‐SNE) and clustered using the Louvain method. *(Right) Known* cell fates can be mapped to clusters by examining localization of characteristic markers. Clusters correspond to (1) myeloblasts, (2) monoblasts, (3) lymphoid cells (4) stem and progenitors, and (5) erythroblasts

Accordingly, the most common set of techniques for single‐cell analysis are clustering methods, which typically seek to find distinct groups in the data and associate distinct cell fates with these groups. Figure 4 shows an example of clustering of single‐cell data using the widely employed Louvain clustering technique. Before we consider some specific clustering methods, it is important to note, although it is not often explicitly said, that the connection between data clusters and cell fates is motivated by the theory above. More precisely, in the presence of “noise” (which could be both due to technical measurement error, and/or biologically functional fluctuations in expression levels) theory predicts that clusters in the data will form around attractors of the deterministic dynamics. Typically in experimental studies, clusters are assumed to be related to fixed points of the underlying dynamics. However, this decision is made for reasons of parsimony, not biology. In fact, theory predicts that other topological structures could also appear if the underlying dynamics admit attractors with a more complex geometry (e.g., noisy closed “loops” in expression space would correspond to limit cycles in the underlying dynamics). In practice, however, we do not yet have the tools to search for such structures in the highly noisy data that arise from single‐cell experiments, hence the current focus on clustering. This may change in the future though, and it is likely that advances in single‐cell profiling methods that produce more finely detailed, and less noisy data, in combination with developments in topological data analysis (Carlsson, 2009) will soon allow us to identify more complex dynamics from single‐cell profiling data.

Unsupervised clustering and community detection methods generally aim to identify partitions in data (Blondel, Guillaume, Lambiotte, & Lefebvre, 2008; Girvan & Newman, 2002; Lloyd, 1982). As such, they can be used to deconstruct the map from experimentally sampled cell states, *x*_*i*_ ∈ *X*, to discrete cell fates, *y*_*i*_ ∈ *Y* (Figure 1). There are now a variety of different methods in use for this purpose: each method makes different assumptions about the data, and different assumptions about the underlying dynamics. For example, *k*‐means clustering is a widely used method that seeks to minimize the within‐cluster sum of squares, and so prefers clusters that are of similar size and approximately “spherical” (Kiselev, Andrews, & Hemberg, 2019; Lloyd, 1982). From the dynamical systems perspective, this corresponds to an assumption that the basins of attraction of the cell fate attractors are of similar size and simple geometry. Because this is a rather strong assumption, which may not be the case, there have been attempts to refine *k*‐means clustering for single‐cell data (Grün et al., 2015). Hierarchical clustering (another widely used generic clustering method), by contrast, allows for more complicated cluster geometries, and additionally arranges clusters within a nested hierarchy (Carlsson, 2009; Maimon & Rokach, 2005; Sibson, 1973; Ward Jr, 1963). Allowing for such hierarchical ordering is often a benefit: for example, in cases where cell fates can be decomposed into subtly different subtypes each with slightly different molecular signatures corresponding to different functional biases, hierarchical clustering can provide insight not only into individual cell fates but also the relationships between them (Zeisel et al., 2015). Yet, it is not always the case that cell fates can be easily arranged in a hierarchy, and so hierarchical clustering methods are not always appropriate. To circumvent these issues, a range of alternative methods are also used. Currently, the Louvain method is perhaps the most popular technique for clustering single‐cell data, since it is able to detect clusters of arbitrary structure without making assumptions on how clusters are related, so is generally better suited to dissecting the structure of complex cell fate landscapes (Blondel et al., 2008; Butler, Hoffman, Smibert, Papalexi, & Satija, 2018; Duò, Robinson, & Soneson, 2018; Freytag, Tian, Lönnstedt, Ng, & Bahlo, 2018; Girvan & Newman, 2002; Stuart et al., 2019; Wolf, Angerer, & Theis, 2018). This flexibility is achieved by considering pairwise distances between cells independently and restricting attention to the *k*‐nearest neighbors of each cell, rather than considering distances between all pairs of cells. Doing so allows the data to be represented as a graph, in which each cell is a node and edges represent nearest neighbor relationships. Once constructed, this graph can be partitioned into sets of densely connected nodes (cells), independently of the geometry of how these sets are arranged in the expression space, *X*.

## 5 VISUALIZATION AND DIMENSIONALITY REDUCTION

In addition to clustering, a range of other data analysis methods are used to deconstruct the landscape of cell fate. Most commonly, clustering is performed in conjunction with dimensionality reduction.

The data that results from single‐cell profiling experiments are typically high‐dimensional, and therefore hard to interpret intuitively. There has, therefore, been considerable effort to apply a range of dimensionality reduction methods to single‐cell data (Becht et al., 2019; Ding, Condon, & Shah, 2018; Van Dijk et al., 2018). As with clustering, the rationale for this effort is not just pragmatic. Because functional associations between genes give rise to correlations in expression patterns, it is expected that important structural features of the data will be lower dimensional than the space in which the data may be collected (i.e., the dimension of the expression space *X*).

The basic method for dimensionality reduction is principal components analysis (PCA) (Hotelling, 1933; Pearson, 1901). PCA is a linear method that essentially seeks to translate and rotate coordinates such that the first‐derived coordinate (or principal component, PC) captures as much variability in the data as possible and each successive PC is orthogonal to the last and captures as much of the remaining variability as possible. PCA is quick and easy to implement, and can be surprisingly informative. For example, because each PC is a linear combination of underlying variables (e.g., the gene expression levels), the results of PCA can be easily interpreted and interrogated for biological meaning. For this reason, it is widely used. However, because PCA is a linear method and biology is inherently (and often strongly) nonlinear, it is inherently limited in dissecting single‐cell data. For this reason, there have been a number of attempts to derive alternative methods that are able to better capture biology's nonlinearities.

A common, characteristic feature of these methods is that they recognize that to properly understand expression data we need to refine our notion of “distance” between cells. To see why this is important it is helpful to return to Waddington's landscape and note that to traverse a path between two points on the landscape, we are not free to travel directly “as the crow flies” but rather the path(s) that we can take, and therefore, the distance between points is constrained by the structure of the landscape itself. Importantly, the length of the shortest path, or geodesic, between two points may therefore be very different from the Euclidean distance between the points. This discrepancy may not be significant for points that are close to each other on the landscape but can be acute when considering the distance between points that are far apart, because the geodesic may involve traversing over multiple hills and through multiple valleys. For this reason, the most popular nonlinear dimensionality methods for single‐cell expression data, such as t‐distributed stochastic neighbor embedding (t‐SNE), and uniform manifold approximation and projection (UMAP) seek to balance local and global notions of distance (Becht et al., 2019; Grün et al., 2015; Hinton & Roweis, 2003; Klein et al., 2015; Maaten & Hinton, 2008). The results of these methods are typically two‐dimensional “maps” of expression patterns that capture the essential structure of the data yet represent it in a two‐dimensional form that is easier to analyze visually. Often dimensionality reduction methods are used in combination with clustering methods to show how cell fates are arranged relative to one another. Figure 4 shows the typical output of such an analysis.

## 6 TRAJECTORY INFERENCE

The methods described so far aim to identify different cell fates from data and represent relationships between them. Such analysis is appropriate whenever the cell fate dynamics are at equilibrium. However, many experiments investigate cell populations as they transition from one fate to another. Because they probe the transition between fates, such dynamics are out‐of‐equilibrium. Examples of this kind of experiment include monitoring the dynamics of the induction of pluripotency in vitro, or the dynamics of stem cell lineage differentiation in vivo (Dahlin et al., 2018; Nestorowa et al., 2016; Paul et al., 2015; Takahashi & Yamanaka, 2006).

The theory described above also provides a simple framework to interpret these experiments. First, note that the field ***F***(***x***) in Equation (1) that describes the cellular dynamics will typically depend upon a set of bifurcation parameters ***μ*** that may change over time (for example, due to environmental changes). Importantly, at certain critical points, known as bifurcation points, ***F***(***x***;***μ***) will change its topology, resulting in the loss or gain of attractor states. If a developmental or experimental process tunes a subset of bifurcation parameters such that when one attractor state loses stability another gains stability, a cell will move along a trajectory in expression space and a fate change may occur. Biologically, these bifurcation parameters often correspond to the expression levels of various different morphogens. For example, it is well known that a set of critical values in the level of the morphogen Sonic Hedgehog determines the stability different cell fates in the ventral neural tube Balaskas et al. (2012).

There is much interest in better understanding the typical trajectories that are followed by cells during such cell fate transitions. Importantly, because current single cell profiling methods are destructive, it is not possible to continuously monitor individual cells as they change fate, and so there has been considerable interest in developing computational methods that are able to infer trajectories from samples of cells taken periodically as they undergo a transition. These methods are known as trajectory inference and in recent years, trajectory inference has become a rapidly growing field—for instance, over 70 distinct methods were identified in a recent review (Saelens, Cannoodt, Todorov, & Saeys, 2019). Inferred trajectories are particularly interesting from a biological perspective because they can be used to assign an ordering to sampled cell states, enabling the dynamics of individual molecular species to be determined, thereby helping to dissect the molecular mechanisms that drive cell fate changes (Aibar et al., 2017; Trapnell et al., 2014).

Cell fate trajectories are often inferred by finding a directed network of cell state “milestones” that provide a skeleton that captures the essential topology of trajectories being examined (Saelens et al., 2019). Different methods make different assumptions concerning the structure of this network. Most commonly it is assumed to have a tree‐like structure which can contain a series of branches, but not cycles (Saelens et al., 2019; Street et al., 2018). In this case, putative branch points (at which a cell fate “choice” is made, and different cells may adopt divergent fates) can also be identified and the molecular changes that occur when cell fates diverge can be investigated. From a dynamical systems perspective such branch points are interesting because they occur whenever there is a bifurcation in the underlying dynamics. Thus, trajectory inference methods can, in principle, allow indirect exploration of bifurcation points. Typically, this is currently done in a relatively ad hoc way; however, we anticipate that future methods will allow us to better understand the nature of the bifurcations that regulate cell fate choices directly from experimental data.

## 7 CONCLUSIONS

In this primer, we have introduced some notions from dynamical systems theory and discussed how they can be used to better understand cell fate dynamics both in theory and practice. This is an area that has its roots in pioneering work by Waddington, Delbrück, Kauffman and others, yet is still an issue that is at the forefront of modern cell biology. Indeed our understanding of the relationships between cell states, fates, and the dynamics of fate acquisition is still in its infancy (Clevers et al., 2017). As we develop increasingly sophisticated experimental ways to interrogate cells as they move between states, there is a concurrent need to refine, revise, and improve our theoretical understanding of cell fate dynamics. Indeed, to properly understand modern experimental data, a sound theoretical foundation is needed. We anticipate that the coming years will see ever‐closer integration of theory and experiment in this area that will, in turn, yield a deeper understanding of cell fate dynamics.

## CONFLICT OF INTEREST

The authors have declared no conflicts of interest for this article.

## AUTHOR CONTRIBUTIONS

**Michael Casey**: Conceptualization; writing‐original draft, review, and editing. **Patrick Stumpf**: Data curation; resources; visualization. **Ben MacArthur**: Conceptualization; supervision; visualization; writing‐original draft, review, and editing.

## RELATED WIREs ARTICLE

https://doi.org/doi.org/10.1002/wsbm.1163
